# Monolithic Opto‐Acoustic Synesthetic Transduction of Color and Sound in a Single Chiral Liquid Crystal Elastomer

**DOI:** 10.1002/smll.74150

**Published:** 2026-06-11

**Authors:** Ji Yoon Park, In Pyo Hong, Seohyun Woo, Jun Hyuk Shin, Sang Hyun Han, Hak Jun Yang, Su Seok Choi

**Affiliations:** ^1^ Department of Electrical Engineering Pohang University of Science and Technology (POSTECH) Pohang South Korea; ^2^ Graduate School of Semiconductor Technology Pohang University of Science and Technology (POSTECH) Pohang South Korea

**Keywords:** chiral liquid crystal elastomer, monolithic, opto‐acoustic, synesthetic

## Abstract

Chiral liquid crystal elastomers (CLCEs) have been widely explored for structural color modulation via tunable photonic bandgaps, yet their functionality has remained largely limited to optical responses. Here, we introduce an opto‐acoustic chiral liquid crystal elastomer actuator (OA‐CLCEA) that redefines CLCEs as intrinsically multimodal transducers, enabling simultaneous color modulation and sound generation within a single soft material layer. Under a static DC electric field, Maxwell stress induces thickness compression and reduces the chiral pitch, producing continuous, wavelength‐resolved structural color tuning, while an AC electric field drives membrane vibration to generate audible sound across 20 Hz–20 kHz. Notably, the intrinsic DC–AC cross‐term in the Maxwell stress enables simultaneous yet decoupled control of optical wavelength and acoustic frequency, allowing genuine orthogonal modulation within a single material system. Unlike conventional multimodal platforms based on heterogeneous architectures, the OA‐CLCEA functions as both a photonic modulator and an acoustic emitter through a unified electromechanical mechanism. This monolithic design simplifies device architecture while enabling compactness and electrical programmability, and establishes a material‐level opto‐acoustic transduction strategy. These results further position CLCEs as an unexplored platform for active acoustic emission and provide a scalable route toward reconfigurable multimodal devices and synesthetic human–machine interfaces.

## Graphical abstract

Monolithic cholesteric liquid crystal elastomers simultaneously generate color and sound under electrically programmed stimuli. Low‐frequency electric fields modulate the helical pitch to produce tunable structural colors, while high‐frequency excitation induces membrane vibration for acoustic emission. Frequency‐domain separation allows independent and simultaneous electrical control of optical and acoustic outputs within a single elastomeric device.

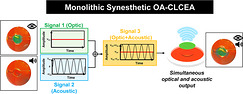

## 1 Introduction

Recent advances in artificial sensory systems have accelerated the development of multimodal electronic interfaces that integrate multiple human sensory modalities for more efficient interaction with external environments [1, 2, 3, 4]. Moving beyond single‐sensory processing, synesthetic devices that convert a single stimulus into multiple sensory outputs have emerged as a promising platform for human‐machine interfaces (HMIs). Given the dominant roles of vision and hearing in human perception [5, 6], electronic systems capable of synchronously delivering visual color information and auditory cues offer clear advantages in information density, perceptual immersion, and communication efficiency [7]. Crucially, realizing these advantages requires independent and continuous control over optical color wavelength and acoustic frequency.

From the perspective of free and continuous control of true optical wavelengths, nanophotonic structural color platforms based on selective photonic bandgap (PBG) control of chiral liquid crystals (CLCs) have attracted considerable attention [8, 9, 10, 11]. Unlike conventional pigment‐based color generation [12] or emission‐based color generation [13, 14], which is inherently constrained by the fixed absorption or emission spectra, typically limited to primary colors, the precisely engineered nanoscale PBG of CLCs offers a high degree of freedom in wavelength modulation [10, 15, 16, 17]. Moreover, because the helical pitch of CLCs responds to external stimuli such as temperature [18, 19], electric fields [15, 20, 21], and light [22, 23, 24, 25, 26], the selective optical wavelength can be dynamically tuned, enabling access to a broad, continuously addressable color gamut with high color purity from a single device without relying on conventional primary color mixing schemes. When incorporated into elastomeric polymer networks, CLCs form chiral liquid crystal elastomers (CLCEs), a representative class of soft photonic materials that integrate selective structural color based on PBG properties with mechanical elasticity [27, 28, 29, 30, 31]. Through direct modulation of their helical pitch, CLCEs exhibit rapid and reversibly controllable wavelength changes under mechanical deformation [32, 33, 34, 35]—such as stretching, compression, or bending—making them highly attractive for biomimetic smart skins [36] and mechanically reconfigurable photonic platforms with fine wavelength tunability [37, 38, 39, 40]. Despite these advantages, prior studies on CLCEs have predominantly focused on their optical functionalities, while the broader potential of CLCEs to function as direct, active acoustic emitters has remained largely unexplored.

Meanwhile, efforts to integrate acoustic and optical functions have attracted increasing attention; however, significant limitations remain. Other approaches to single‐film opto‐acoustic devices suffer from intrinsic coupling between optical responses and the driving frequency or require additional phosphors or emissive materials to achieve multicolor operation [41, 42, 43, 44, 45, 46]. Consequently, although the integration of acoustic and optical functionalities has been demonstrated, their independent control remains limited, and the material system is therefore effectively a multi‐component composite rather than a truly monolithic platform. By contrast, previously reported synesthetic devices often rely on heterogeneously stacked or hybrid architectures, such as photonic hydrogels integrated with separate dielectric elastomer actuators (DEAs) [47]. Although such systems can enable simultaneous color and sound generation, their architecture inherently limits the realization of a truly monolithic platform and constrains independent, continuously tunable control of optical and acoustic outputs. As a result, a genuinely single‐material, monolithic opto‐acoustic synesthetic platform has yet to be realized.

Here, we overcome these intrinsic limitations by introducing an intrinsically single‐material, monolithic synesthetic platform in which both optic and acoustic outputs are electrically induced within a single chiral liquid crystal elastomer (CLCE) layer. To the best of our knowledge, we demonstrate for the first time that CLCEs—traditionally regarded solely as tunable photonic materials—can also function as active acoustic media through electrically induced mechanical vibration. To realize this concept, we devise a single‐layer opto‐acoustic CLCE actuator (OA‐CLCEA), consisting of a CLCE film sandwiched between optically transparent, highly stretchable ionic‐gel electrodes that ensure uniform electric‐field application without mechanically constraining the deformation of the elastomer. Under a static electric field, electrostatic attraction between the electrodes compresses the CLCE along the thickness direction, reduces its helical pitch, and induces an electric‐field‐dependent blue shift of the reflected wavelength, enabling electrochromic color modulation. In parallel, when an alternating current (AC) signal within the human audible frequency range (20 Hz–20 kHz) is applied, the CLCE undergoes direct periodic vibration at the driving frequency and emits vivid sound, exhibiting a distinct electro‐acoustic response.

Importantly, this architecture enables independent and decoupled control of optical color and acoustic sound within a single monolithic CLCE layer: static electric fields govern color tuning via helical pitch compression, whereas AC signals independently define acoustic output through vibration frequency and amplitude. Consequently, the reflected wavelength and acoustic frequency constitute two orthogonal and continuously tunable control parameters, fundamentally distinguishing our platform from previous synesthetic devices and enabling a genuinely single‐material system with independent and continuous control over both optical and acoustic responses.

## 2 Results and Discussion

**FIGURE 1:**
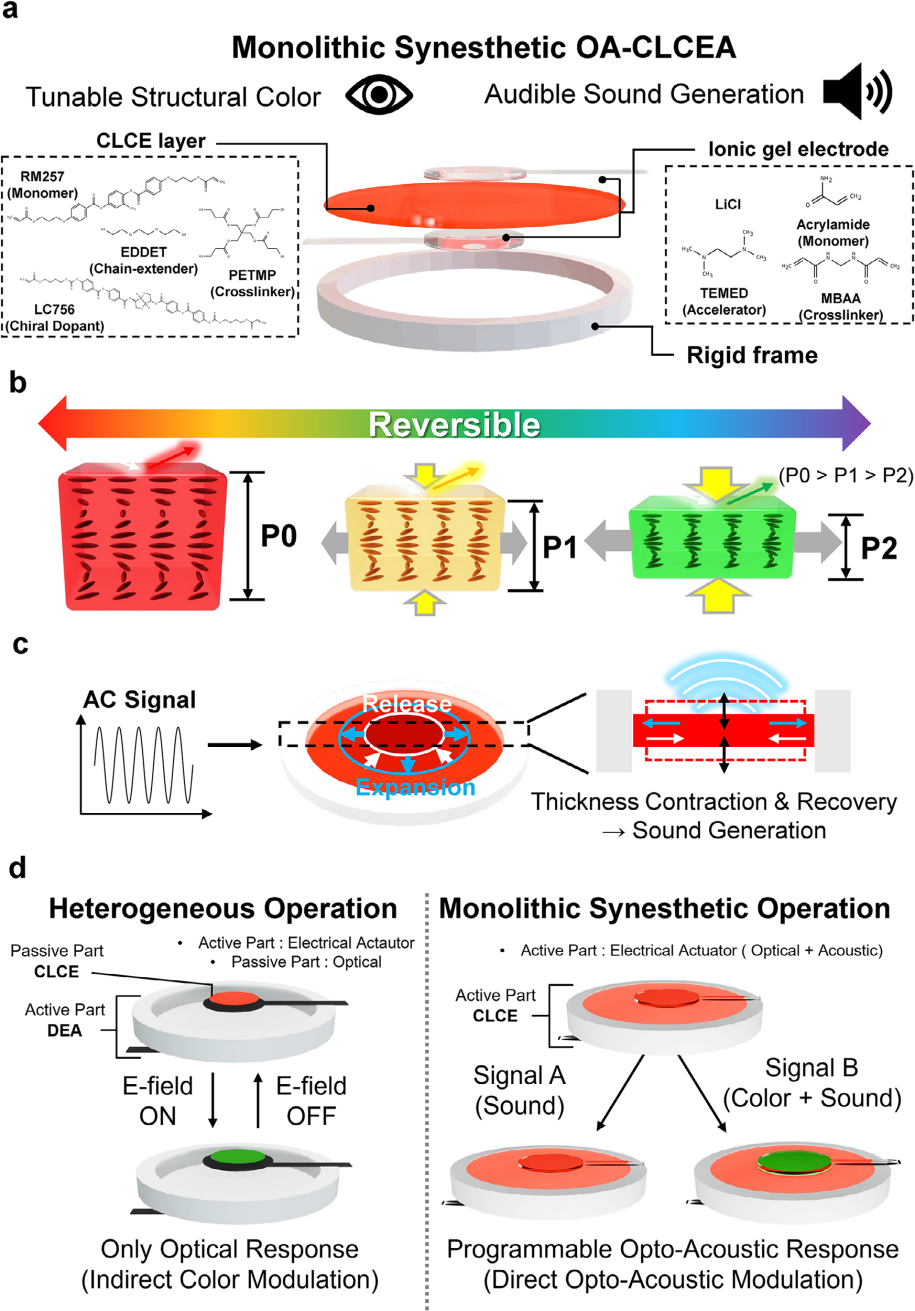
Design and operational principles of the monolithic synesthetic OA‐CLCEA. (a) Schematic illustration of monolithic device architecture and chemical components. (b) Mechanism of reversible structural color tuning. (c) Mechanism of acoustic signal generation under an AC electric field. (d) Comparison between heterogeneous operation and the proposed monolithic synesthetic operation.

Figure 1 illustrates the structure of the OA‐CLCEA and its monolithic synesthetic operations. The device architecture and constituent materials are shown in Figure 1a. The OA‐CLCEA consists of a CLCE film serving as the active layer, assembled in a sandwich configuration between stretchable and optically transparent ionic gel electrodes. The mechanical properties of the synthesized CLCE, characterized by stress‐strain measurements, are provided in Figure S1. Optical transparency of the electrodes is essential for visualizing the electric‐field‐induced color change in the CLCE [48, 49], and the optical transmittance of the ionic gel electrode is provided in Figure S2. Further characterization of the ionic gel electrode, including its frequency‐dependent impedance, stretchability, and electrostatic actuation mechanism, is provided in Figure S3. In parallel, the same CLCE layer sandwiched between the optically transparent ionic‐gel electrodes undergoes electrically induced oscillatory vibration, thereby acting as an active actuator that generates acoustic sound. To suppress undesired out‐of‐plane deformation, the CLCE film was biaxially pre‐stretched by 5% and fixed within a rigid acrylic frame. Detailed fabrication procedures and material compositions are described in the Experimental section and Table S1.

Figure 1b schematically illustrates the general reversible pitch‐tuning mechanism of CLCEs under mechanical deformation. When a CLCE film is mechanically stretched in the in‐plane direction, the nearly incompressible elastomer contracts along the thickness direction, reducing the helical pitch formed along the film thickness. Consequently, the reflection wavelength shifts toward shorter wavelengths (blue shift), and this color change is fully reversible upon removal of the applied stimulus. Note that the intrinsic structural color of CLCEs originates from selective color wavelength reflection of PBG, which can be described by the de Vries equation. Here, the central wavelength of the PBG (*λ*_c_) is expressed as

(1) $$\lambda_{c}=n_{avg}\;\cdot p$$

where n_avg_ is the average refractive index of the CLCE and p is the helical pitch, defined as the period over which the liquid crystal mesogens complete one full rotation [50].

Figure 1c illustrates the dynamic mechanism by which the OA‐CLCEA generates sound under the application of an alternating current (AC) signal. In contrast to the mechanical stretching case shown in Figure 1b, electrical actuation of the OA‐CLCEA is driven by Maxwell stress acting directly across the CLCE thickness between the top and bottom ionic‐gel electrodes. This direct Maxwell‐stress‐induced compression results in thickness reduction of the CLCE layer. Under periodic AC electric field therefore induces oscillatory thickness contraction and recovery of the CLCE layer, accompanied by lateral deformation due to elastomer incompressibility. This periodic out‐of‐plane (z‐axis) membrane vibration compresses and rarefies the surrounding air, generating pressure waves that propagate as acoustic signals directly from the CLCE without the need for additional sound‐generating components. As a result, direct opto‐acoustic transduction can be realized, whereby electrically induced membrane vibration for sound and wavelength‐level structural color modulation are co‐localized within a single CLCE layer.

Although electrical operation has been demonstrated across various liquid crystal elastomers (LCEs)—from high‐speed electrostatic multimodal actuation in conventional LCEs [51] to optical wavelength modulation in CLCEs [52, 53, 54]—these prior approaches have primarily targeted either mechanical or optical responses, leaving the potential of CLCEs as direct, active acoustic emitters largely unexplored. Moreover, these approaches have largely relied on heterogeneous configurations in which CLCEs are indirectly coupled to additional actuating DEAs parts. Figure 1d compares the operating concepts of conventional electrically driven CLCE devices with the monolithic synesthetic OA‐CLCEA proposed in this work. Conventional CLCE‐based devices primarily employ CLCEs as passive optical layers integrated on active DEAs, enabling only static optical structural color changes induced indirectly by the electric‐field‐driven actuation of the DEAs. Notably, the use of CLCEs themselves as active acoustic emitters has remained unexplored. In contrast, the OA‐CLCEA developed here enables programmable opto‐acoustic responses within a single CLCE layer by tailoring the waveform of the applied electrical signals, allowing not only independent acoustic output but also direct and simultaneous visual and auditory responses. Beyond their conventional role as tunable photonic materials, OA‐CLCEA is revealed here as an electrically addressable opto‐acoustic transducer, enabling genuinely monolithic synesthetic operation without auxiliary actuators or heterogeneous integration.

**FIGURE 2:**
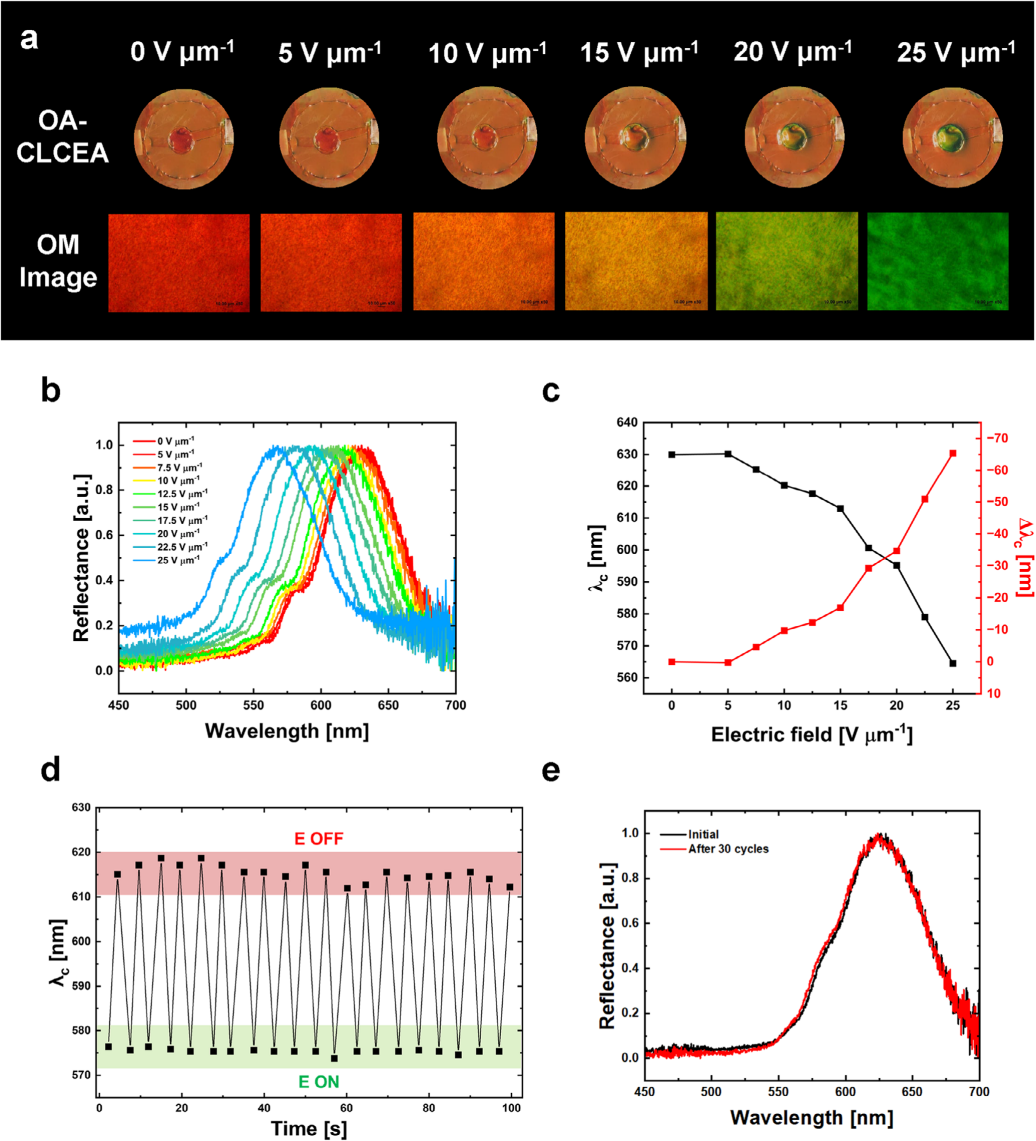
Characterization of the electrochromic performance of the OA‐CLCEA. (a) Device photographs and optical microscopic (OM) images of the OA‐CLCEA under various electric field strengths. (b) Reflection spectra showing a gradual blue shift of the reflection band with increasing electric field. (c) Central wavelength (λ_c_) and corresponding wavelength shift (Δλ_c_) as a function of the applied electric field. (d) Repeatability of the OA‐CLCEA under cyclic electrical deformation. (e) Reflectance spectra of the OA‐CLCEA before and after 30 cycles of electrical actuation.

To verify the monolithic synesthetic functionality of a single‐layer OA‐CLCEA, we first examined its electrically driven color‐tuning characteristics. Figure 2 presents the electrochromic behavior of the OA‐CLCEA. Upon application of an electric field to direct OA‐CLCEA, the electrode‐covered region exhibited a continuous color change from red to green, which was clearly observed under optical microscopy (Figure 2a; Figure S4). To quantitatively analyze this color variation, the reflectance spectra of the OA‐CLCEA were measured under different direct current (DC) electric field strengths (Figure 2b). As the applied electric field increased, the reflectance spectrum gradually shifted toward shorter wavelengths, with the central reflection wavelength decreasing from approximately 630–565 nm (Δλc = −65 nm), as summarized in Figure 2c. The hysteresis behavior of the central reflection wavelength during electrical loading–unloading cycles was further evaluated (Figure S5). This electrically induced wavelength shift originates from Maxwell stress generated by electrostatic attraction between the top and bottom ionic‐gel electrodes under a quasi‐static DC electric field. The resulting stress compresses the CLCE layer along the thickness direction, reduces the chiral pitch, and consequently shifts the reflected wavelength toward shorter values. This wavelength tuning corresponds to static modulation of the central reflection wavelength as a function of the applied DC electric field, thereby defining the maximum achievable color‐tuning range of the OA‐CLCEA. The transient structural‐color response was further measured under a step electric‐field input with off–on–off states of 15, 25, and 50 s, respectively (Figure S6). Based on the 10%–90% and 90%–10% wavelength transitions, the rise and fall times of the central wavelength were determined to be 6.09 and 1.37 s, respectively.

To evaluate the operational stability of the OA‐CLCEA, the dynamic response of the central selective reflection wavelength was monitored under continuous electrical operation for 100 s (Figure 2d). The device was driven by a sinusoidal electric field with an amplitude of 25 V µm^−^^1^ at a frequency of 0.1 Hz. Throughout the test, the OA‐CLCEA exhibited stable and fully reversible operation without observable optical or mechanical degradation. Notably, the measured mechanical response frequency was 0.2 Hz, corresponding to twice the applied electrical driving frequency. This frequency‐doubling behavior confirms that the deformation is governed by Maxwell stress, which depends on the magnitude of the electric field rather than its polarity. A representative real‐time visualization of the electrochromic color switching under the same driving conditions is provided in Video S1. To further assess the device reliability, the reflectance spectra were measured before and after 30 cycles of electrical actuation under identical driving conditions (Figure 2e). The two spectra are nearly identical, confirming that the OA‐CLCEA retains its photonic band structure and mechanical integrity during repeated electrical operation.

These results confirm that the single‐layer OA‐CLCEA achieves stable, reversible, and fatigue‐free electrochromic color tuning through direct Maxwell‐stress‐driven actuation of the CLCE itself, establishing robust monolithic optical functionality without reliance on heterogeneous dielectric actuators and providing a solid foundation for subsequent opto‐acoustic synesthetic operation.

**FIGURE 3:**
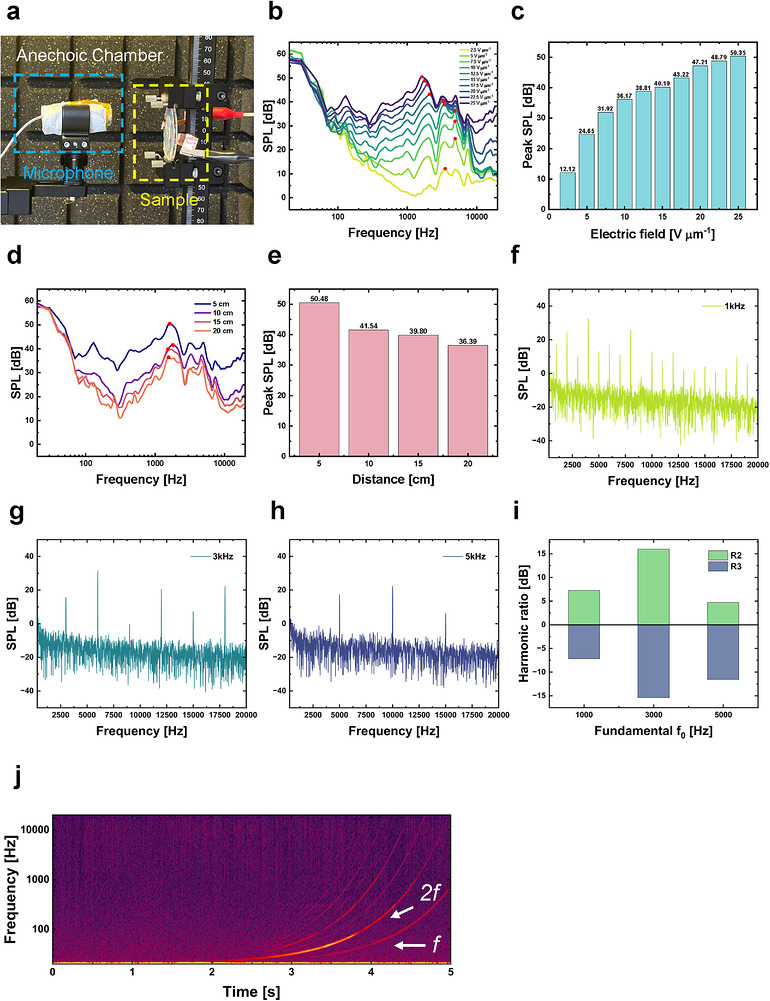
Electro‐acoustic characteristics of the OA‐CLCEA. (a) Photograph of the experimental setup used to evaluate the electro‐acoustic output. (b) SPL spectra as a function of the applied electric‐field amplitude. (c) Extracted peak SPL values for different applied electric‐field amplitudes. (d) SPL spectra as a function of the microphone distance. (e) Extracted peak SPL values as a function of the measurement distance. (f–h) Frequency response of the OA‐CLCEA at (f) 1, (g) 3, and (h) 5 kHz, showing distinct fundamental peaks and their associated harmonic components. (i) Harmonic distortion characteristics of the OA‐CLCEA, showing the second‐harmonic ratio (R2) and third‐harmonic ratio (R3) at 1, 3, and 5 kHz. (j) Spectrogram of the OA‐CLCEA, visualizing the frequency‐dependent acoustic emission characteristics.

Building on the demonstrated monolithic electrochromic functionality, Figure 3 presents the electroacoustic characteristics of the OA‐CLCEA under electrical excitation within the human audible‐frequency range (20 Hz–20 kHz). To validate the electrically driven sound‐generation mechanism, acoustic emission from the OA‐CLCEA was characterized in an anechoic chamber, as illustrated in Figure 3a. The device was mounted on a fixed support, and a calibrated microphone was positioned to record the acoustic signals emitted directly from the OA‐CLCEA. The electroacoustic response was quantified by measuring the sound pressure level (SPL) as a function of electrical driving conditions (Figure 3b–e). A representative demonstration of the acoustic output during an AC frequency sweep from 20 Hz to 20 kHz is provided in Video S2. Based on the background noise spectrum of the anechoic chamber, the SPL components below 100 Hz are likely dominated by background noise rather than device‐generated acoustic output (Figure S7). As shown in Figure 3b, the SPL increases with the magnitude of the applied electric field, which is attributed to the enhanced membrane displacement amplitude under stronger Maxwell stress, resulting in higher acoustic pressure. The peak SPL values, marked by red circles in Figure 3b, were further extracted and quantitatively compared as a function of the applied electric field in Figure 3c. The corresponding acoustic output characteristics, including peak frequency, peak SPL, background noise, and SNR under different applied electric fields, are summarized in Table S2. In addition, the transient acoustic response was evaluated under a step electric‐field input, revealing rapid turn‐on and turn‐off dynamics with a 10%–90% rise time of 0.76 ms and a 90%–10% fall time of 3.00 ms after baseline‐noise correction (Figure S8).

Furthermore, SPL measurements performed while varying the distance between the OA‐CLCEA and the microphone in 5 cm increments (Figure 3d) reveal a systematic increase in SPL as the microphone is positioned closer to the device, confirming direct sound emission from the vibrating CLCE membrane. The corresponding peak SPL values, marked by red circles in Figure 3d, are summarized in Figure 3e, further confirming the distance‐dependent acoustic output of the OA‐CLCEA. The detailed distance‐dependent acoustic output characteristics are provided in Table S3.

To further analyze the acoustic response of the OA‐CLCEA, the SPL spectra were carefully measured at fundamental frequencies of 1, 3, and 5 kHz under an AC electric field of 21.8 V µm^−^^1^ with zero DC electric field (Figure 3f–h). Distinct peaks were observed at each fundamental frequency, accompanied by harmonic components at integer multiples of the fundamental frequency. These results confirm that the OA‐CLCEA is driven by Maxwell stress, which depends quadratically on the applied electric field, thereby producing unavoidable nonlinear responses that manifest as harmonic distortion. Although these spectra include higher‐order harmonic peaks, the present analysis focuses on the second and third harmonics as representative low‐order nonlinear components. The higher‐order harmonics are attributed to the combined effects of Maxwell‐stress‐induced nonlinear electromechanical coupling, membrane vibration modes, and viscoelastic deformation of the CLCE layer. To quantitatively compare the relative strength of the second and third harmonic components with respect to the fundamental, the harmonic ratio was defined. The second harmonic ratio (R2) and third harmonic ratio (R3) are expressed as

(2) $$R2=20log_{10}\frac{p_{2f0}}{p_{f0}}$$

(3) $$R3=20log_{10}\frac{p_{3f0}}{p_{f0}}$$

where p_f0_, p_2f0_, and p_3f0_ represent the sound pressures at the fundamental, second harmonic, and third harmonic frequencies, respectively. The analysis revealed that R2 exhibited positive values at all driving frequencies of 1, 3, and 5 kHz, whereas R3 showed negative values (Figure 3i). This indicates that the second harmonic component consistently dominates over the third harmonic component in the acoustic output of the OA‐CLCEA.

Consistent with the observation that the second harmonic dominates under specific driving frequencies, the broadband electroacoustic response of the OA‐CLCEA was further investigated by applying a logarithmic frequency sweep from 20 Hz to 20 kHz over a duration of 5 s (Figure 3j). The resulting acoustic output is presented as a spectrogram obtained from Fourier transform–based time–frequency analysis, in which brighter colors indicate higher sound pressure levels (SPL). As indicated by the white arrows in Figure 3j, the lower‐frequency trajectory corresponds to the fundamental component (*f*), whereas the brighter upper trajectory corresponds to the second harmonic (2*f*). The logarithmic sweep generates a fundamental frequency component that follows an exponential trajectory along the time axis, accompanied by clearly resolved harmonic components at integer multiples of the fundamental frequency. Notably, across most of the audible frequency range, the second harmonic exhibits a higher amplitude than the fundamental component, confirming that the nonlinear acoustic response of the OA‐CLCEA—originating from Maxwell‐stress‐driven actuation—is not limited to discrete excitation frequencies but is consistently manifested throughout the entire audible spectrum. From a synesthetic control perspective, this intrinsic nonlinearity is not merely a source of harmonic distortion but constitutes an additional degree of freedom, enabling rich and programmable coupling between optical wavelength modulation and acoustic spectral content within a single CLCE layer. In contrast to conventional opto‐acoustic systems that seek to suppress nonlinearity, the OA‐CLCEA deliberately exploits this inherent nonlinear response to expand the synesthetic design space, allowing complex sound generation while preserving continuous wavelength‐level color tunability.

**FIGURE 4:**
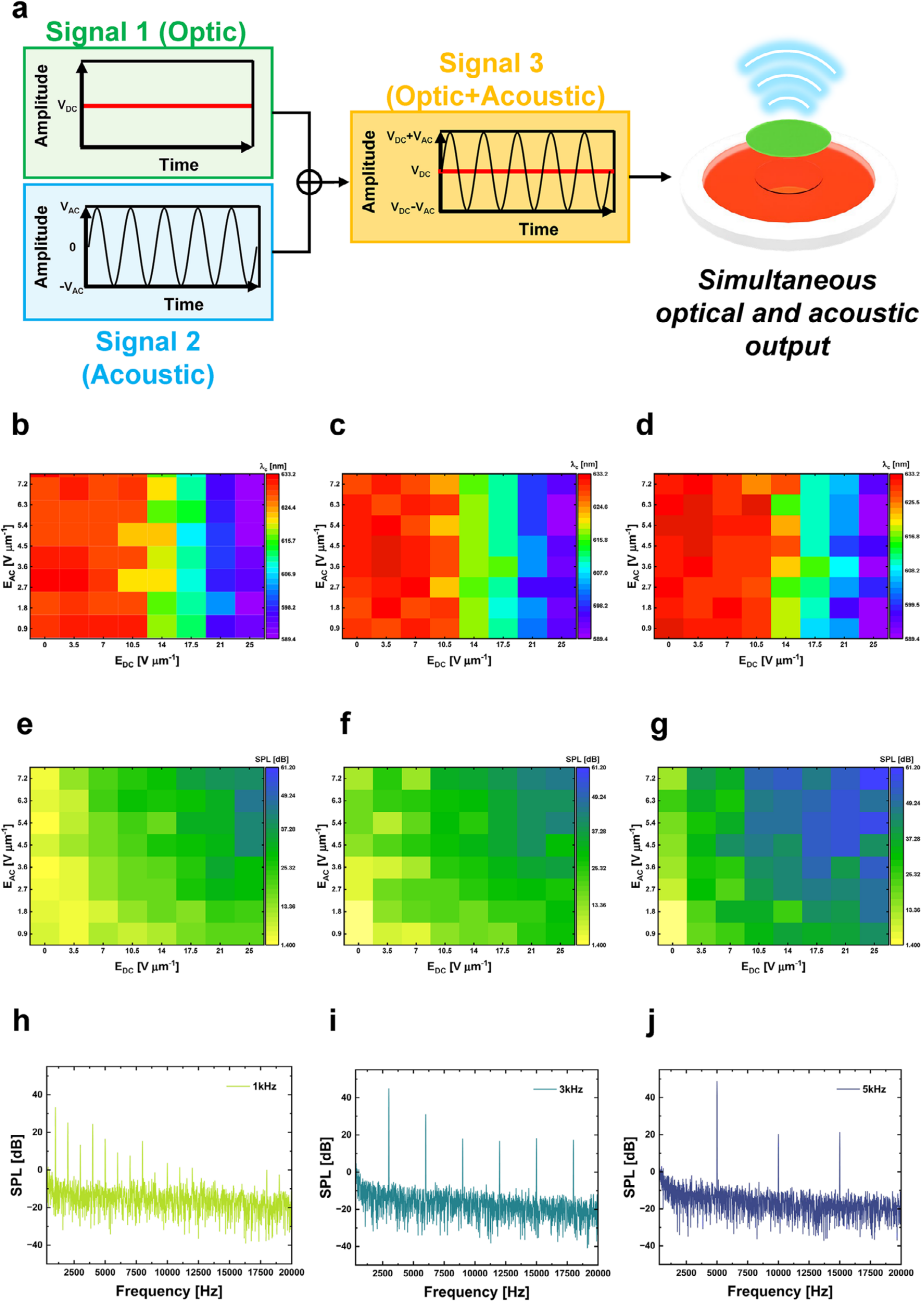
Multimodal opto‐acoustic response of the OA‐CLCEA under combined DC‐AC excitation. (a) Schematic illustration of the signal integration method for OA‐CLCEA driving. (b–d) Structural‐color response maps as a function of *E*_DC_ and *E*_AC_ under sinusoidal excitation at (b) 1 kHz, (c) 3 kHz, and (d) 5 kHz, showing blue‐shifts of the reflected wavelength. (e–g) Sound pressure level (SPL) maps as a function of *E*_DC_ and *E*_AC_ measured at (e) 1, (f) 3, and (g) 5 kHz, showing a clear increase in SPL with increasing *E*_DC_ under all driving frequencies. (h–j) Frequency spectra of the OA‐CLCEA driven at (h) 1, (i) 3, and (j) 5 kHz under combined DC‐AC excitation, showing pronounced fundamental peaks with reduced harmonic components.

While the OA‐CLCEA generates audible sound under single AC excitation, the associated color shift becomes negligible at high driving frequencies because the time‐averaged thickness deformation is insufficient to induce effective pitch modulation (Figure S9 and Video S3). Beyond this single‐signal operation, true synesthetic functionality was demonstrated by simultaneously applying direct current (DC) and alternating current (AC) electrical signals, enabling concurrent color modulation and sound generation within the same CLCE layer (Figure 4). As illustrated in the driving waveform in Figure 4a, the total applied electric field, E(t), is defined as follows:

(4) $$E\;\left(t\right)=E_{DC}+E_{AC}\sin\left(\omega t\right)$$

where *E*_DC_ and *E*_AC_ represent the DC and AC components of the applied electric field, respectively. In this configuration, the constant *E*_DC_ induces the optical response associated with color change, while the time‐varying *E*_AC_ drives the acoustic response. When the combined signal is applied, the OA‐CLCEA functions as a multimodal device capable of simultaneously controlling both color and sound (Video S4).

Figure 4b–d presents the optical response of the OA‐CLCEA as a function of the AC and DC electric‐field components (*E*_AC_ and *E*_DC_) at driving frequencies of 1, 3, and 5 kHz. The AC field amplitude (*E*_AC_) was varied from 0.9 to 7.2 V µm^−^^1^ under DC‐biased operation, with higher fields above 8.1 V µm^−^^1^ causing electrical breakdown of the device; accordingly, 7.2 V µm^−^^1^ was set as the upper operational limit. The DC field (*E*_DC_) was applied in the range of 0–25 V µm^−^^1^. Across all three frequency conditions, increasing *E*_DC_ induced a pronounced blue shift of the central reflection wavelength of the OA‐CLCEA. In contrast, variations in *E*_AC_ exerted only a minor influence on the wavelength shift compared to the DC component. A similar trend was observed at higher driving frequencies, as shown in Figure S10.

These results indicate that optical color modulation in the OA‐CLCEA is predominantly governed by the DC electric field, whereas the AC component exerts only a minor influence on the optical response. This behavior arises because the static DC field generates a sustained Maxwell stress that compresses the CLCE membrane along the thickness direction, reduces the chiral pitch, and consequently shifts the selective reflection wavelength toward shorter values. In contrast, the AC field oscillates in time and contributes negligibly to the time‐averaged stress; instead, it primarily induces dynamic membrane vibration, which gives rise to acoustic emission rather than optical wavelength modulation.

Figure 4e–g presents the acoustic response of the OA‐CLCEA measured at driving frequencies of 1, 3, and 5 kHz under identical combinations of *E*_AC_ and *E*_DC_. Across all three frequency conditions, the sound pressure level (SPL) increases monotonically with increasing *E*_DC_, a trend that is consistently observed at higher driving frequencies as well (Figure S11). This DC‐induced enhancement of SPL originates from the electric‐field dependence of the Maxwell stress. As detailed in Note S1, the Maxwell stress responsible for acoustic emission contains a cross‐term between the DC and AC components, which provides the dominant contribution to sound generation. Consequently, a higher *E*_DC_ establishes a stronger electrostatic bias that amplifies the mechanical response of the CLCE to the applied AC signal. In this context, *E*_DC_ acts as a tunable gain parameter for acoustic output while simultaneously governing optical color modulation, underscoring the dual functionality of the OA‐CLCEA in exploiting orthogonal electrical field components to drive multimodal synesthetic responses.

Figure 4h–j shows the acoustic response spectra of the OA‐CLCEA under driving frequencies of 1, 3, and 5 kHz by applying a combined electric field of *E*_AC_ 14.5 V µm^−^^1^ and *E*_DC_ 7.3 V µm^−^^1^. In all cases, the fundamental frequency component was clearly observed and compared to the results without a DC component (Figure 3d–f), the relative amplitude of the harmonic components was reduced. This indicates that the *E*_DC_ modifies the bias state of the membrane, thereby making the cross term with the AC signal more dominant, which in turn suppresses the harmonic distortion originating from the nonlinear terms of Maxwell stress.

This observation is further supported by the second‐ and third‐harmonic ratios (R2 and R3) presented in Figure S12. Under combined DC–AC excitation, both R2 and R3 exhibit negative values, indicating that the relative contribution of the harmonic components decreases and the acoustic output becomes increasingly dominated by the fundamental frequency. This behavior corroborates that the *E*_DC_ shifts the operating point of the membrane into a regime where the nonlinear terms of the Maxwell stress are effectively suppressed, thereby reducing harmonic distortion.

Physically, the DC–AC cross‐term in the Maxwell stress provides the OA‐CLCEA with an intrinsic gain mechanism, whereby a static electric‐field bias linearly amplifies the mechanically driven acoustic response without perturbing the time‐averaged optical deformation state. This clear separation between static and dynamic electromechanical contributions enables simultaneous yet decoupled control of optical wavelength modulation and acoustic emission within a single material system. Unlike conventional opto‐acoustic platforms that rely on separate actuators or intrinsically coupled driving conditions—often resulting in trade‐offs between optical and acoustic performance—the OA‐CLCEA exploits the inherent DC–AC coupling of Maxwell stress to achieve orthogonal and independently programmable control of color and sound within a genuinely monolithic architecture.

**FIGURE 5:**
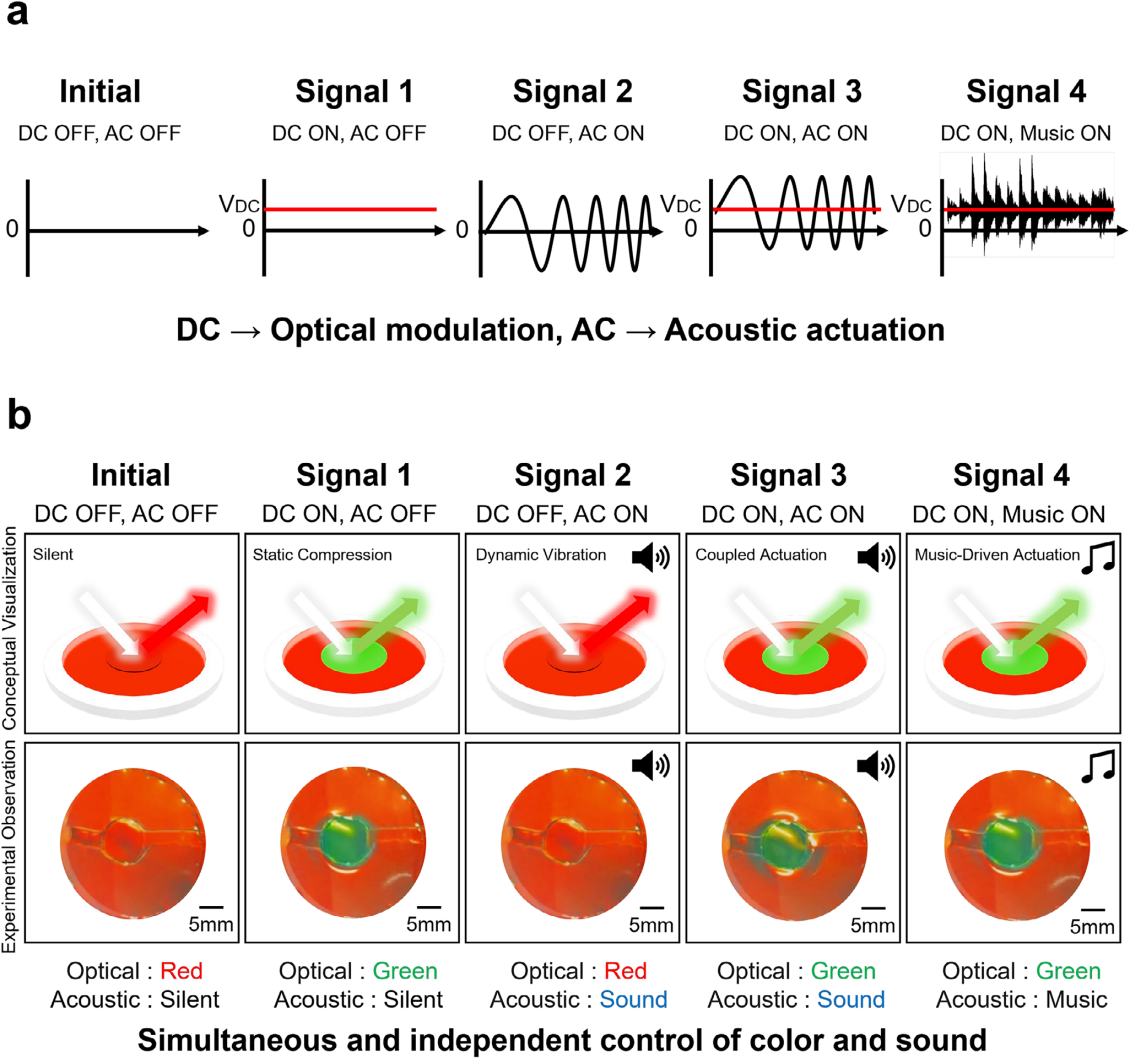
Multimodal opto‐acoustic responses of the OA‐CLCEA under different electrical input signals. (a) Schematic of driving signal waveforms, including an initial state followed by four programmed electrical inputs: Signal 1 (*E*_DC_ only), Signal 2 (*E*_AC_ only, frequencies corresponding to the sixth octave, C6‐B6), Signal 3 (*E*_DC_ and *E*_AC_ combined), and Signal 4 (*E*_DC_ with AC music signal). (b) Conceptual visualizations (top) and experimental photographs (bottom) showing the corresponding opto‐acoustic outputs. Starting from the initial silent red state, the device demonstrates four distinct actuation modes: Static Compression, Dynamic Vibration, Coupled Actuation, and Music‐Driven Actuation, highlighting the simultaneous and independent control of color and sound. All scale bars are 5 mm.

Figure 5 demonstrates the integrated opto‐acoustic synesthetic operation of the OA‐CLCEA in response to diverse electrical input signals, including real‐time actual musical excitation. Figure 5a schematically illustrates the driving waveforms corresponding to an initial state and four distinct operation modes. In the initial state, both DC and AC components are turned off (*E*_DC_ = 0, *E*_AC_ = 0). Signal 1 (*E*_DC_ = 25 V µm^−^^1^, *E*_AC_ = 0) applies a static DC bias only, Signal 2 (*E*_DC_ = 0, *E*_AC_ = 7.3 V µm^−^^1^) applies a single‐tone AC excitation only, Signal 3 (*E*_DC_ = 25 V µm^−^^1^, *E*_AC_ = 7.3 V µm^−^^1^) combines DC and single‐tone AC components, and Signal 4 (*E*_DC_ = 25 V µm^−^^1^, *E*_AC_ = 7.3 V µm^−^^1^) applies a complex music waveform under DC bias. Signals 2 and 3 employ single‐tone AC excitations corresponding to sixth‐octave musical notes (C6–B6), whereas Signal 4 applies a complex music waveform derived from variations of *“Twinkle, Twinkle, Little Star.”* Under this signal configuration, the DC component deterministically governs optical color wavelength modulation of the OA‐CLCEA through static Maxwell stress, while the AC component independently drives mechanical vibration and sound emission. This demonstration confirms that the OA‐CLCEA can simultaneously render dynamic visual color changes and audible music within a single CLCE layer, establishing a fully integrated opto‐acoustic synesthetic platform capable of real‐time, programmable multimodal expression.

Figure 5b presents conceptual visualizations (top) alongside experimental photographs (bottom) of the corresponding opto‐acoustic responses for the initial state and each operation mode. All experiments were performed using an OA‐CLCEA initially exhibiting red structural color. In the initial state, the device remains optically red and acoustically silent. Upon application of Signal 1 (static compression mode), the device shows a pronounced blue shift from red to green, originating from DC‐induced thickness compression and the associated reduction in helical pitch; in the absence of an AC component, no acoustic output is observed. By contrast, under Signal 2 (dynamic vibration mode), the absence of a DC field preserves the initial red reflection state, while the applied AC signal induces mechanical vibration of the CLCE, generating audible sound in the sixth‐octave frequency range. When DC and AC components are simultaneously applied in Signal 3 (coupled actuation mode) using the same AC frequency range, the OA‐CLCEA exhibits concurrent optical and acoustic responses: the DC field induces a green reflection, while the AC signal produces the target acoustic tones. Notably, the fundamental acoustic frequencies remain invariant with respect to the DC‐induced reflection color, whereas the acoustic amplitude scales naturally with the magnitude of the applied DC field (Figure S13 and Video S5), further confirming the independent yet synergistic control of optical and acoustic outputs within a single CLCE layer. Finally, under Signal 4 (Music‐Driven Actuation mode), a complex music waveform (Variations of *“Twinkle, Twinkle, Little Star”*) is superimposed onto the *E*_DC_. The detailed music‐to‐electrical driving signal conversion and acoustic signal‐processing workflow are provided in Figure S14. In this mode, the device maintains the green reflection state while successfully reproducing the music. The corresponding spectrogram confirms that the OA‐CLCEA reproduces the temporal and spectral characteristics of the applied music signal under DC‐biased excitation (Figure S15 and Video S6).

Collectively, these results demonstrate that the selective reflection color and acoustic output of the OA‐CLCEA can be independently and simultaneously controlled within a single device through tailored electrical input signals. Beyond simple tonal excitation, the successful rendering of complex musical waveforms underscores the potential of the OA‐CLCEA as a genuinely single‐material, multimodal interface capable of delivering integrated visual and auditory feedback for synesthetic human–machine interaction.

## 3 Conclusion

In this study, we report a genuinely monolithic synesthetic opto‐acoustic platform based on a single chiral liquid crystal elastomer layer, termed the OA‐CLCEA, in which color wavelength modulation and audible sound generation are electrically realized within one soft photonic material. Unlike conventional multimodal systems that rely on stacked optical and acoustic modules or heterogeneous composite architectures, the OA‐CLCEA enables independent and orthogonal control of optical wavelength and acoustic frequency within a single material layer, thereby achieving true monolithic synesthetic multimodality while substantially simplifying device structure and fabrication. The operation of the OA‐CLCEA is governed by Maxwell stress‐mediated electromechanical coupling. A static DC electric field induces sustained thickness compression of the CLCEs, reduces the chiral pitch, and enables continuous electrochromic color tuning, whereas an AC electric field drives periodic membrane vibration to generate sound. Crucially, the intrinsic DC–AC cross‐term in the Maxwell stress provides a built‐in electromechanical gain mechanism that allows simultaneous yet decoupled modulation of optical and acoustic responses. This separation of static and dynamic electromechanical contributions enables genuine orthogonal control of color and sound within a single material system, overcoming the fundamental coupling and architectural limitations of previous opto‐acoustic and synesthetic devices. Beyond single‐tone excitation, the OA‐CLCEA reproduces complex audio waveforms, including music, under DC‐biased operation while maintaining stable, wavelength‐level color modulation, demonstrating practical and fully integrated synesthetic functionality at the device level.

For practical implementation, reducing the driving voltage remains an important future direction. Since the electric field is given by *E*  =  *V*/*d*, optimizing the CLCE thickness is a direct route to lowering the required voltage. A thinner CLCE layer can achieve the same electric field at a lower applied voltage, although this must be balanced with dielectric breakdown stability and mechanical robustness. In addition, lowering the elastic modulus through CLCE network design, optimizing pre‐stretching and device geometry, improving ionic‐gel electrode conductivity, improving dielectric permittivity, and using mechanical amplification or resonance‐based acoustic operation may further reduce the operating voltage while preserving simultaneous optical and acoustic functionality.

In conclusion, by redefining CLCE as active acoustic emitters, this work establishes a new materials‐level strategy for compact, multimodal electronic interfaces. These results position the OA‐CLCEA as a new class of single‐material opto‐acoustic transducer and identify CLCE as an unexplored platform for active acoustic emission. This approach opens new opportunities for compact multimodal interfaces across applications including displays, wearable electronics, smart skins, and soft robotic systems, while providing a general framework for expanding synesthetic functionality in soft electronic and photonic materials.

## 4 Experimental Section

### 4.1 Synthesis of CLCE

Diacrylate reactive mesogen 1,4‐bis‐[4‐(3‐acryloyloxypropyloxy) benzoyloxy]‐2‐methylbenzene (RM257, GrandinChem), and the chiral reactive mesogen (3R,3aS,6aS)‐hexahydrofuro [3,2‐b]furan‐3,6‐diyl bis(4‐(4‐((4‐(acryloyloxy) butoxy) carbonyloxy) benzoyloxy) benzoate) (LC756, BASF) were dissolved into toluene (Sigma‐Aldrich) and heated at 80°C for 10 min to obtain a homogeneous solution. After cooling the solution to room temperature, the thiol crosslinker pentaerythritol tetrakis (3‐mercaptopropionate) (PETMP, Sigma‐Aldrich), the thiol chain extender 2,2′‐(ethylenedioxy)diethanethiol (EDDET, Sigma‐Aldrich), the photoinitiator 2,2‐dimethoxy‐2‐phenylacetophenone (Irgacure 651, Sigma–Aldrich), and catalyst dipropylamine (DPA, Sigma–Aldrich) were added. The mixture was vortexed for 10 min to ensure homogeneity, and air bubbles were removed using a vacuum desiccator. The resulting mixture was bar‐coated onto a conventional OHP film using an applicator with a 220 µm gap at a coating speed of 11.3 mm s^−1^. After solvent evaporation, photopolymerization was carried out in an ultraviolet chamber (CSM1010, AUVCURE) at an intensity of 30 mW cm^−2^ for 5 min, yielding a free‐standing CLCE film with a thickness of approximately 55–60 µm.

### 4.2 Synthesis of Ionic Gel Electrode

A precursor solution was prepared by dissolving 11.7 wt.% acrylamide (AAm, Sigma–Aldrich) and 0.007 wt% N,N'‐methylenebisacrylamide (MBAA, Sigma–Aldrich) into an 88.25 wt.% lithium chloride (LiCl) solution (Sigma–Aldrich). Subsequently, 0.0199 wt.% ammonium persulfate (APS, Sigma–Aldrich) and 0.005 wt.% N,N,N′,N′‐tetrame‐ thylethylenediamine (TEMED, Sigma–Aldrich) were added to the solution. The mixture was gently mixed to ensure homogeneity and then poured into a circular electrode mold with a diameter of 1 cm. Gelation was achieved by heating the mold at 50°C for 2 h to obtain the ionic gel electrode.

### 4.3 Preparation of OA‐CLCEA

A CLCE film was biaxially pre‐stretched by 5% using a biaxial stretching jig to suppress undesired out‐of‐plane deformation and then fixed onto an acrylic rigid frame with a diameter of 3 cm. The 5% pre‐strain was selected to maintain the film in a taut state while minimizing the strain‐induced blue shift of the reflection band, as higher biaxial strains caused a pronounced decrease in the chiral pitch and a corresponding shift in the reflected wavelength (Figure S16). Stress‐relaxation measurements further confirmed that the 5% pre‐strained CLCE film retained a nonzero residual stress after relaxation, supporting maintenance of the taut state (Figure S17). The ionic gel electrodes were gently removed from the mold using tweezers and attached to the top and bottom surfaces of the CLCE layer. Finally, copper tape was affixed to the rigid frame, and the ionic gel electrode pads were placed onto the copper tape to enable electrical connection and voltage application.

### 4.4 Electrochromic and Electro‐Acoustic Characterization

Optical measurements were performed under an unpolarized state using an optical microscope setup consisting of a microscope (BX53M, Olympus), a CMOS image sensor (HAWK‐SCM63, Zootos), and a high‐resolution spectrometer (HR4 Pro, Ocean Insight). For electrochromic characterization of the OA‐CLCEA, the optical microscope setup was connected to an arbitrary function generator (AFC3000C, Tektronix) and a high‐voltage amplifier (609B‐3, Trek). The applied electrical signal was monitored using an oscilloscope (TTBS1000C, Tektronix). A mechanical testing system (Modular Force Stage, Linkam) was used to measure the stress as a function of strain in the CLCE layer. The CLCE layer was stretched at a rate of 500 µms^−1^.

Frequency‐response measurements were performed inside an anechoic chamber. Signal generation and data acquisition of the OA‐CLCEA were controlled using a LabVIEW‐based system, and music signals were delivered through an audio amplifier (MK‐300, Omnitronic). The acoustic output from the OA‐CLCEA was measured using a commercial microphone (4966, Brüel & Kjær (B&K)) and a PXIe‐4464.

## Conflicts of Interest

The authors declare no conflicts of interest.

## Supporting information




**Supporting File 1**: smll74150‐sup‐0001‐SuppMat.docx.

**Supporting File 2**: smll74150‐sup‐0002‐VideoS1.mp4.

**Supporting File 3**: smll74150‐sup‐0003‐VideoS2.mp4.

**Supporting File 4**: smll74150‐sup‐0004‐VideoS3.mp4.

**Supporting File 5**: smll74150‐sup‐0005‐VideoS4.mp4.

**Supporting File 6**: smll74150‐sup‐0006‐VideoS5.mp4.

**Supporting File 7**: smll74150‐sup‐0007‐VideoS6.mp4.

## Data Availability

The data that support the findings of this study are available in the supplementary material of this article.
