# Supplementary material for: Monolithic Opto‐Acoustic Synesthetic Transduction of Color and Sound in a Single Chiral Liquid Crystal Elastomer
Source: Small. 2026 Jun 11;22(42):e74150. doi: 10.1002/smll.74150 (PMC13410848; doi:10.1002/smll.74150)
Supplement: Supplementary file 1 — Supporting File 1: smll74150‐sup‐0001‐SuppMat.docx. [file SMLL-22-e74150-s001.docx]

Supporting Information

Monolithic Opto-Acoustic Synesthetic Transduction of Color and Sound in a Single Chiral Liquid Crystal Elastomer

*Ji Yoon Park^1^, In Pyo Hong^2^, Seohyun Woo^1^, Jun Hyuk Shin^1^, Sang Hyun Han^1^, Hak Jun Yang^1^ and Su Seok Choi^1,2^**

^1^Department of Electrical Engineering Pohang University of Science and Technology (POSTECH)

Pohang, 37673, Korea

^1^ J.Y.P Author 1, S.W. Author 3, J.H.S. Author 4, S.H.H. Author 5, H.J.Y. Author 6 and S.S.C. Corresponding Author

Department of Electrical Engineering, Pohang University of Science and Technology (POSTECH), Pohang, 37673, Korea

^2^I.P.H. Author 2, S.S.C. Corresponding Author

Graduate School of Semiconductor Technology, Pohang University of Science and Technology (POSTECH), Pohang, 37673, Korea

* Corresponding author; E-mail: [choiss@postech.ac.kr](mailto:choiss@postech.ac.kr)

Supplementary Table 1

| Material | RM257 | LC756 | EDDET | PETMP | Irgacure651 | DPA | Toluene |
| --- | --- | --- | --- | --- | --- | --- | --- |
| wt% | 49.55 | 2.36 | 12.07 | 2.72 | 0.33 | 0.16 | 32.81 |

**Table S1. Composition and weight percentages of materials used in the CLCE layer.** The table provides detailed information on the composition and weight percentages of materials used to fabricate the CLCE layer.

Supplementary Table 2

| Electric field  [V μm^-1^] | Peak Frequency [Hz] | Peak SPL [dB] | Background noise [dB] | SNR [dB] |
| --- | --- | --- | --- | --- |
| 2.5 | 3520 | 12.121 | -6.84501 | 18.96601 |
| 5 | 4890 | 24.64728 | -8.54618 | 33.19346 |
| 7.5 | 4860 | 31.91974 | -8.54083 | 40.46057 |
| 10 | 4840 | 36.17403 | -8.52749 | 44.70152 |
| 12.5 | 3410 | 38.8102 | -6.70484 | 45.51504 |
| 15 | 3330 | 40.18947 | -6.77343 | 46.9629 |
| 17.5 | 2090 | 43.22355 | -5.24419 | 48.46774 |
| 20 | 1940 | 47.20742 | -4.78195 | 51.98937 |
| 22.5 | 1760 | 48.79117 | -4.67832 | 53.46949 |
| 25 | 1630 | 50.48247 | -4.58383 | 55.0663 |

**Table S2. Acoustic output characteristics of the OA-CLCEA under different applied electric fields.** The table summarizes the peak frequency, peak sound pressure level (SPL), background noise level, and signal-to-noise ratio (SNR) of the OA-CLCEA as a function of the applied electric field. The SNR was calculated as follows: SNR [dB] = SPL_OA-CLCEA_ [dB] – SPL_noise_ [dB]

Supplementary Table 3

| Distance [cm] | Peak Frequency [Hz] | Peak SPL [dB] | Background noise [dB] | SNR [dB] |
| --- | --- | --- | --- | --- |
| 5 | 1630 | 50.48247 | -4.58383 | 55.0663 |
| 10 | 1810 | 41.54453 | -4.81651 | 46.36104 |
| 15 | 1540 | 39.79862 | -4.33081 | 44.12943 |
| 20 | 1570 | 36.38974 | -4.4744 | 40.86414 |

**Table S3. Distance-dependent acoustic output characteristics of the OA-CLCEA.** The table summarizes the peak frequency, peak sound pressure level (SPL), background noise level, and signal-to-noise ratio (SNR) of the OA-CLCEA measured at different distances under an applied electric field of 25 V μm^-1^. The SNR was calculated using the same equation as in Table S2.

Supplementary Note 1

The acoustic output of the OA-CLCEA is generated by periodic electro-mechanical deformation of the elastomer membrane under an applied electric field. The total applied voltage, $V(t)$, consists of a DC offset voltage $V_{DC}$ and a sinusoidal AC voltage $V_{AC}sin(\omega_{0}t)$ with an angular frequency of $\omega_{0}$:

| $V\left( t \right)=V_{DC}+V_{AC}sin(\omega_{0}t)$ | (1) |
| --- | --- |
|  |  |

The corresponding electric field across the OA-CLCEA layer of thickness $d$ is expressed as

| $E\left( t \right)=\frac{V(t)}{d}=\frac{V_{DC}+V_{AC}sin(\omega_{0}t)}{d}$ | (2) |
| --- | --- |

The Maxwell stress acting on the film is proportional to the square of the electric field:

| $\sigma_{M}=\varepsilon_{0}\varepsilon_{r}{E(t)}^{2}$ | (3) |
| --- | --- |

where $\varepsilon_{0}$ and $\varepsilon_{r}$ are the vacuum and relative permittivity, respectively.

Substituting equation (2) into equation (3) gives

| $\sigma_{M}\left( t \right)=\varepsilon_{0}\varepsilon_{r}\left[ \left( \frac{V_{DC}}{d} \right)^{2}+\frac{2V_{DC}V_{AC}\sin\left( \omega_{0}t \right)}{d^{2}}+\left( \frac{V_{AC}}{d} \right)^{2}{sin}^{2}\left( \omega_{0}t \right) \right]$ | (4) |
| --- | --- |

Using the trigonometric identity ${sin}^{2}\left( \omega_{0}t \right)=\frac{1}{2}(1-\cos\left( 2\omega_{0}t \right))$, equation (4) can be rewritten as

| $\sigma_{M}\left( t \right)=\varepsilon_{0}\varepsilon_{r}\left[ \left( \frac{V_{DC}}{d} \right)^{2}+\frac{\left( V_{AC} \right)^{2}}{2d^{2}}+\frac{2V_{DC}V_{AC}}{d^{2}}\sin\left( \omega_{0}t \right)-\frac{\left( V_{AC} \right)^{2}}{2d^{2}}cos(2\omega_{0}t) \right]$ | (5) |
| --- | --- |
|  |  |

This expression clearly contains three distinct frequency components: a static term (DC), a fundamental term at $f_{0}$ originating from the cross term, and a second-order harmonic term at $2f_{0}$ due to the nonlinear $E^{2}$ dependence of the Maxwell stress.





**Figure S1. Stress-strain curve of free-standing chiral liquid crystal elastomers (CLCEs).**





**Figure S2.** **Optical** **transmittance of ionic gel electrode.** The ionic gel electrode exhibited high optical transparency across the visible spectrum, ensuring minimal optical loss in the OA-CLCEA.


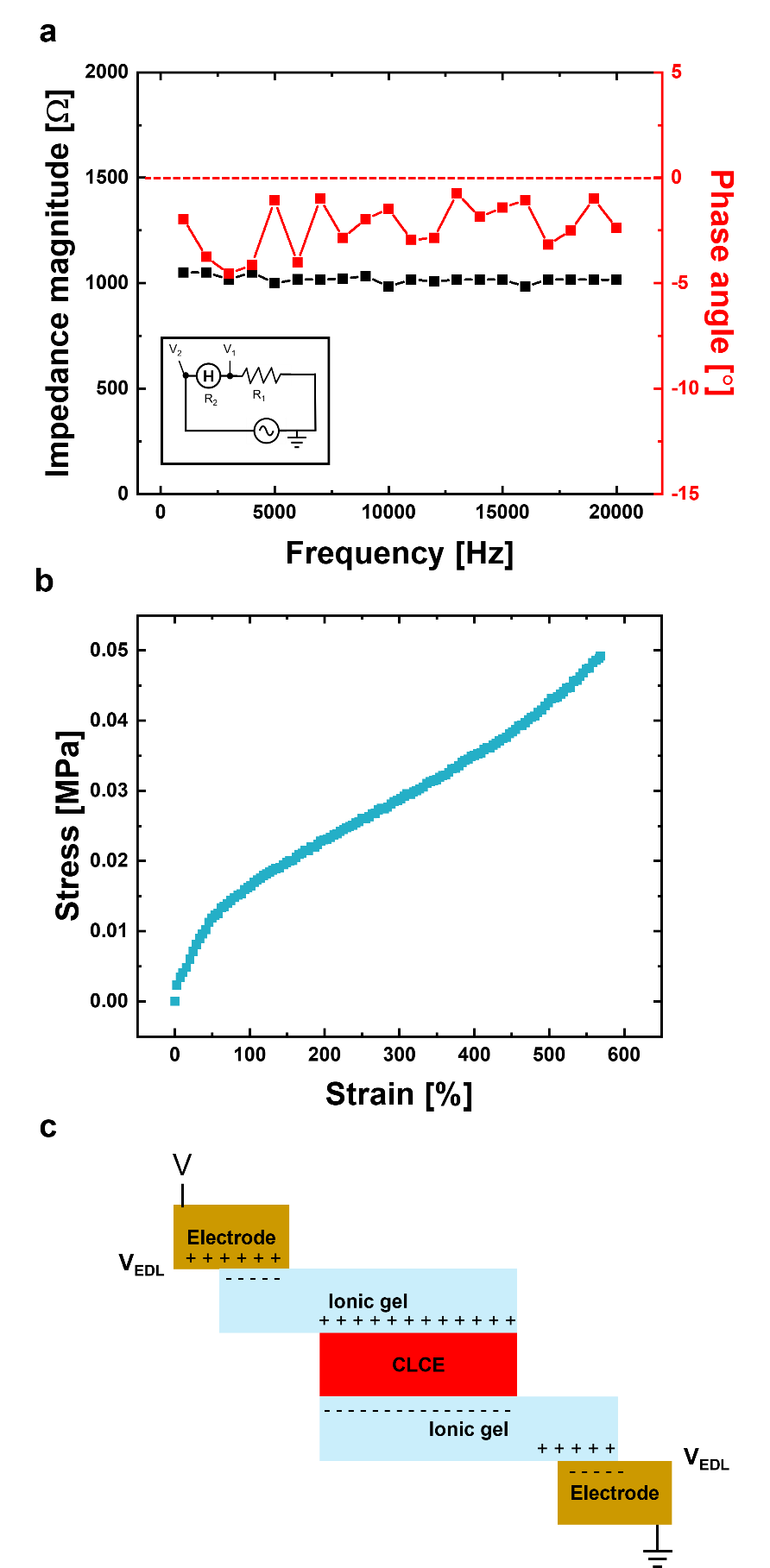


**Figure S3.** **Characterization of the ionic gel electrode and electrostatic actuation mechanism of the OA-CLCEA.** **a)** Frequency-dependent impedance magnitude and phase angle of the ionic gel electrode. **b)** Stress–strain curve of the ionic gel electrode measured at a stretching speed of 500 μm s^-1^. **c)** Schematic illustration of ion accumulation and electrostatic attraction in the OA-CLCEA under an applied voltage.


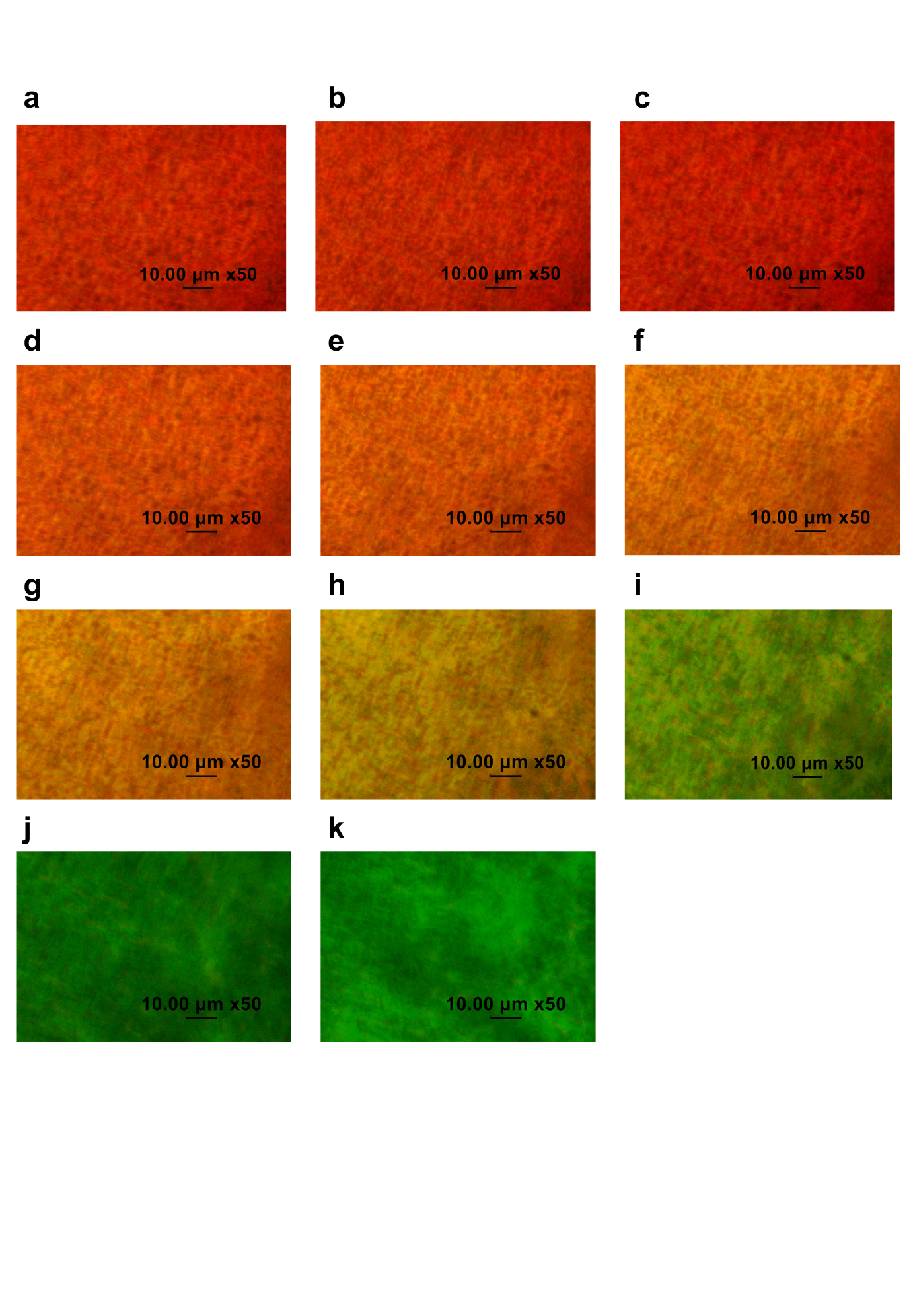


**Figure S4.** **Optical microscopy (OM) images of the opto-acoustic CLCE actuator (OA-CLCEA) under different applied electric fields. a-k)** Reflection-mode OM images of the OA-CLCEA recorded at increasing electric fields from 0 V $\mu$m^-1^ to 25 V $\mu$m^-1^ in increments of 2.5 V $\mu$m^-1^.

**Figure S5. Central wavelength shift and hysteresis behavior of the OA-CLCEA under an applied electric field.** The change in the central wavelength is shown as the electric field varies. The wavelength shift signifies continuous structural color modulation. The pronounced hysteresis observed in the curve reflects the viscoelastic characteristics and the slow relaxation of molecular reorientation of the CLCE.


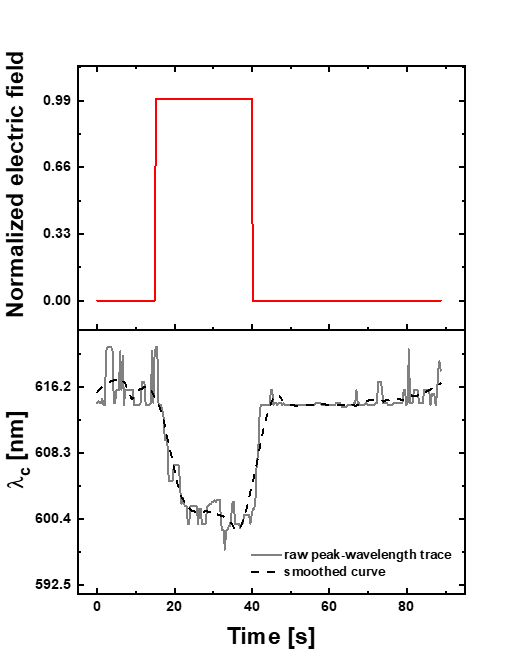


**Figure S6.** **Transient structural-color response of the OA-CLCEA under step electric field input.** Time-dependent center wavelength shift of the OA-CLCEA measured under a step electric field with 15 seconds off, 25 seconds on, 50 seconds off states. The 10% and 90% thresholds were defined from the wavelength difference between the voltage-off state (λ_off_=614.277 nm) and the on-state (λ_on_=600.416 nm), corresponding to a total wavelength shift of 13.861 nm. The extracted 10-90% rise time and 90-10% fall time were 6.09 seconds and 1.37 seconds, respectively.

**
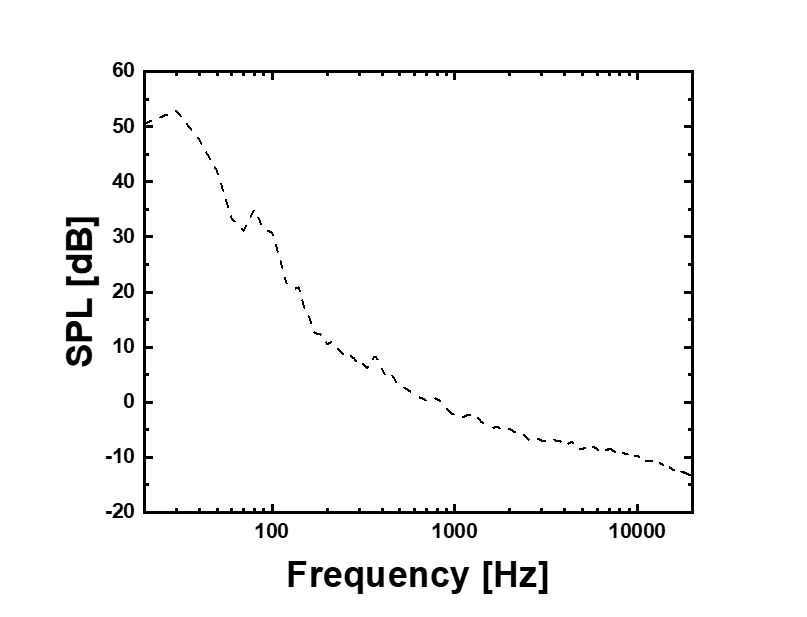
**

**Figure S7. Background noise spectrum of the anechoic chamber.** Sound pressure level (SPL) spectrum measured in the anechoic chamber without OA-CLCEA actuation. The relatively large SPL components below 100 Hz indicate that low-frequency signals in this range are mainly attributed to background noise rather than device-generated acoustic output.

*
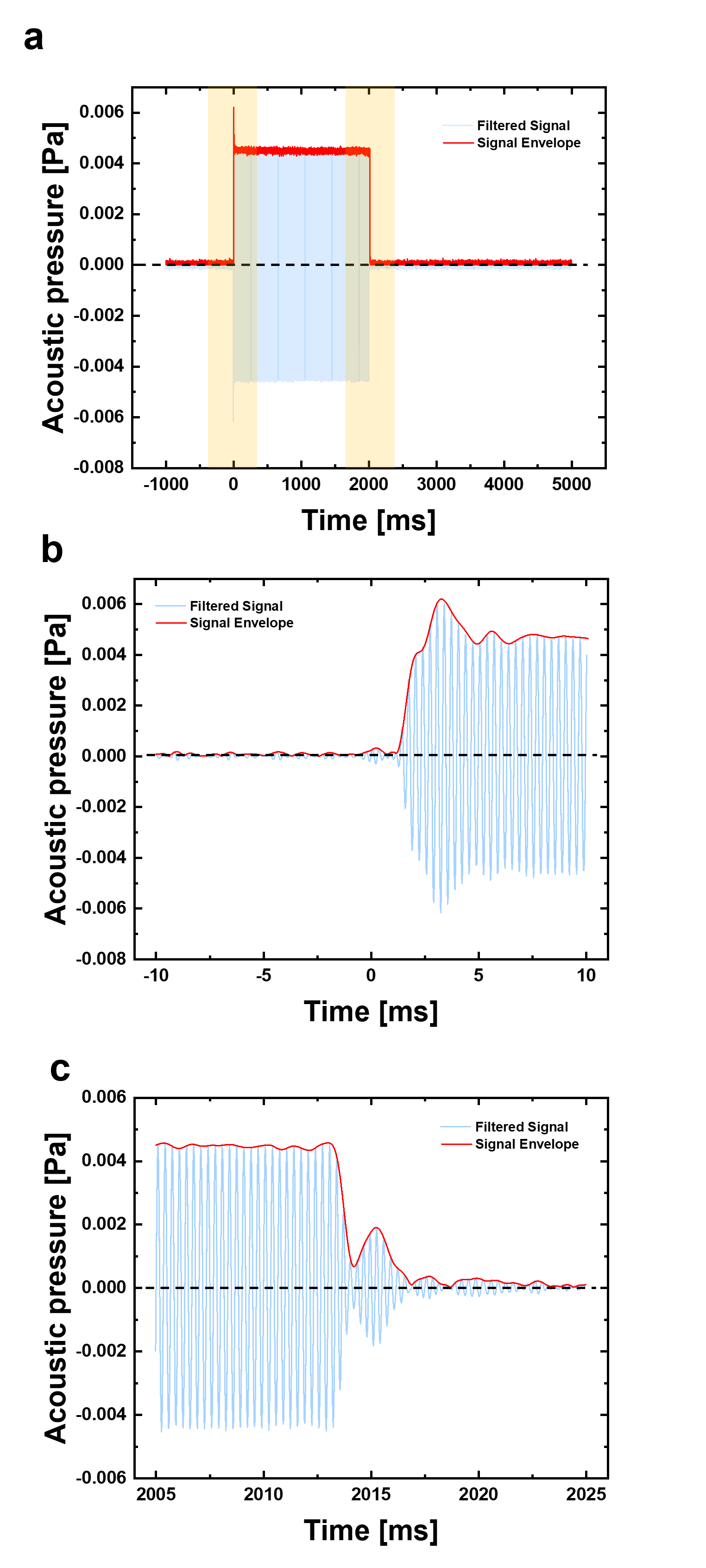
*

**Figure S8. Transient acoustic response of the OA-CLCEA under step electric-field input. a)** Step electric-field input and corresponding acoustic pressure response of the OA-CLCEA. **b)** Magnified turn-on region showing the 10–90% rise time extracted from the acoustic pressure envelope. **c)** Magnified turn-off region showing the 90–10% fall time extracted from the acoustic pressure envelope. The rise and fall times were determined to be 0.76 ms and 3.00 ms, respectively, after considering the pre-trigger baseline noise level.


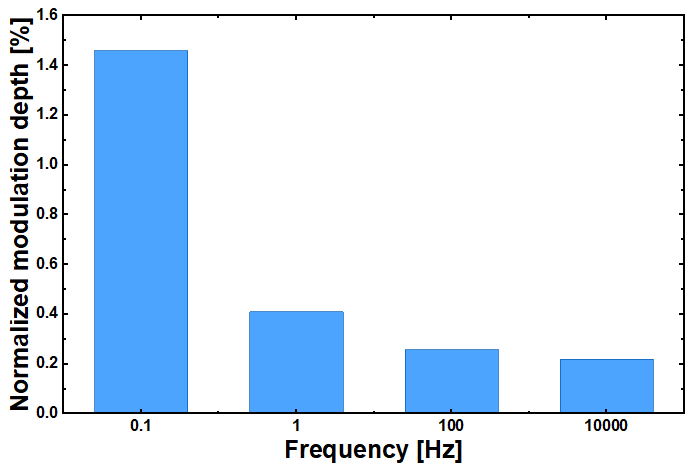


**Figure S9. Frequency‐dependent change in the normalized modulation depth M(f) of the OA-CLCEA.** Bar graph showing the optical response efficiency of the OA-CLCEA under varying AC driving frequencies. The normalized modulation depth is defined as $M\left( f \right)=(\lambda_{p95}-\lambda_{p5})/\lambda_{0}\times100\%$, which quantifies the relative magnitude of the electrically induced color change.


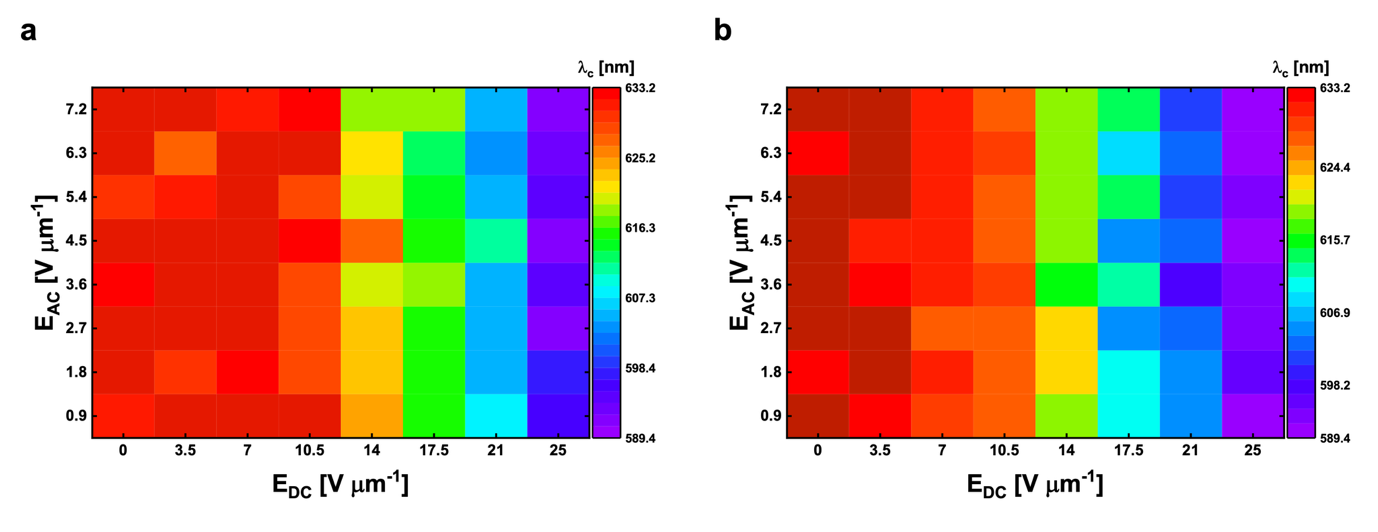


**Figure S10.** **Central wavelength of the OA-CLCEA as a function of AC electric field (E_AC_) and DC electric field (E_DC_) at different driving frequencies.** (a) 10 kHz and (b) 15 kHz.


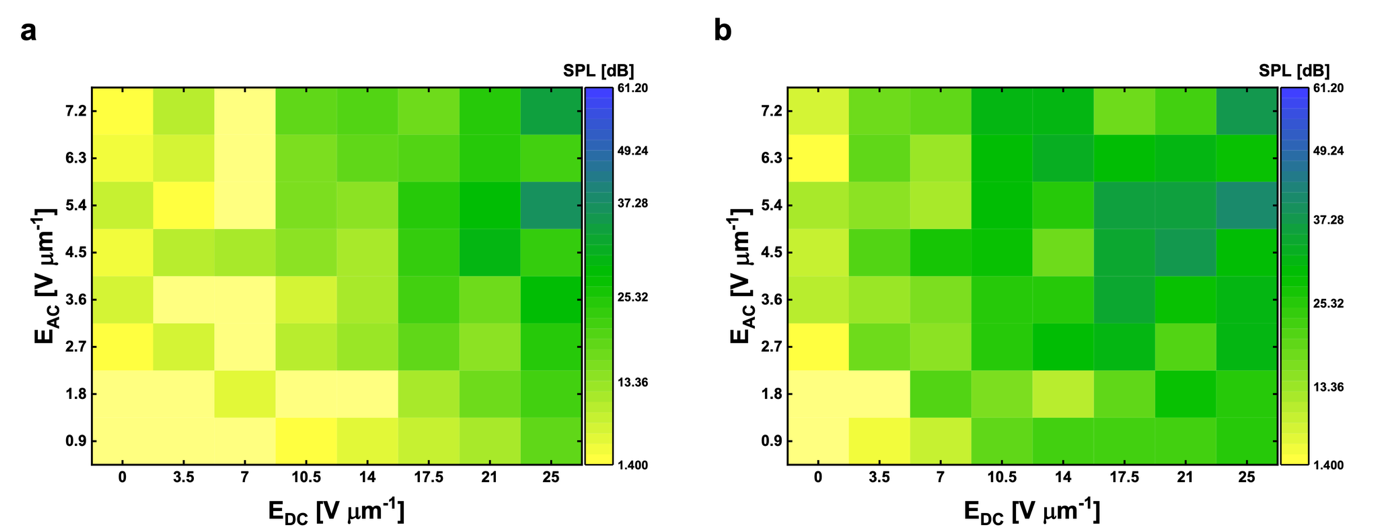


**Figure S11.** **Sound pressure level (SPL) of the OA-CLCEA as a function of AC electric field (E_AC_) and DC electric field (E_DC_) at different driving frequencies.** (a) 10 kHz and (b) 15 kHz. The SPL increases with E_DC_, indicating that the applied E_DC_ enhances the electrostatic attraction and amplifies the acoustic output of the actuator.


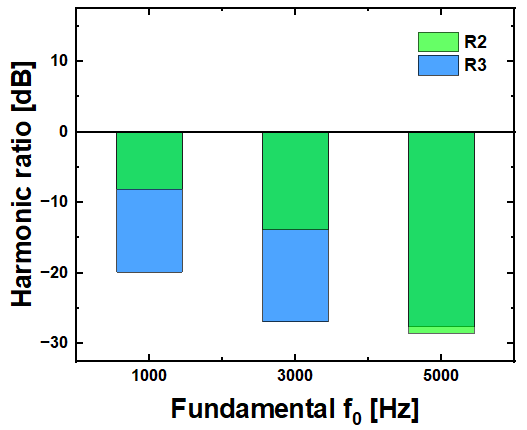


**Figure S12.** **Harmonic distortion ratios of the OA-CLCEA under combined AC and DC excitation.** The OA-CLCEA was driven with an AC electric field (E_AC_) of 14.5 V μm⁻¹ and a DC electric field (E_DC_) of 7.3 V μm⁻¹ at fundamental frequencies of 1, 3, and 5 kHz. Both R2 and R3 exhibit negative values, indicating that the introduction of an E_DC_ enhances the amplitude of the fundamental frequency component while suppressing nonlinear harmonic generation in the acoustic response.


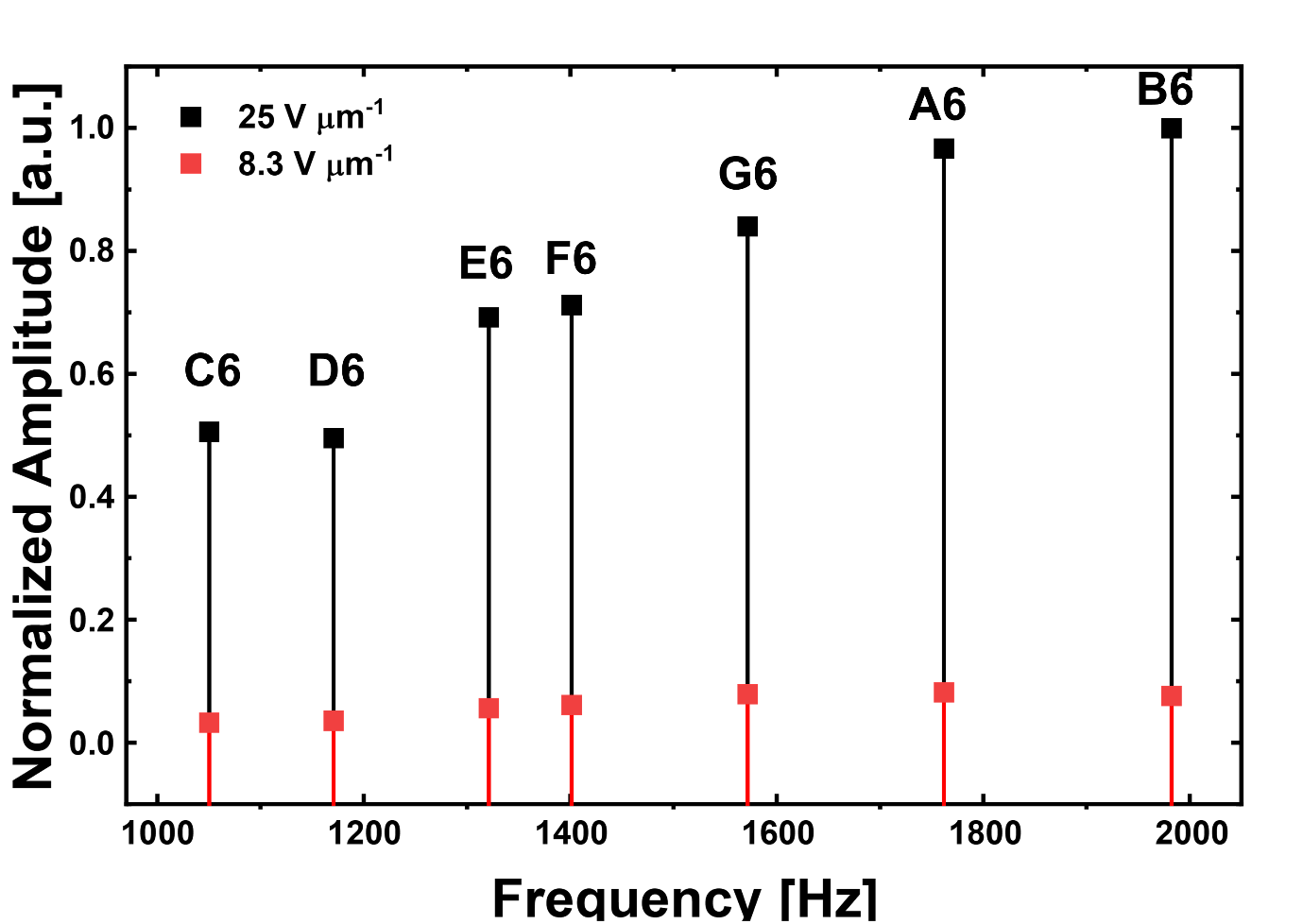


Figure S13. Acoustic output of the OA-CLCEA under different DC electric field states. Both E_DC_ = 8.3 V μm⁻¹ and E_DC_ = 25 V μm⁻¹ conditions, driven with an identical AC electric field amplitude (E_AC_) of 6.7 V μm⁻¹, produced clear acoustic vibrations at the corresponding frequencies of the 6-octave tones (C6-B6), confirming that sound generation is frequency-driven and remains independent of the electrically tuned color state.


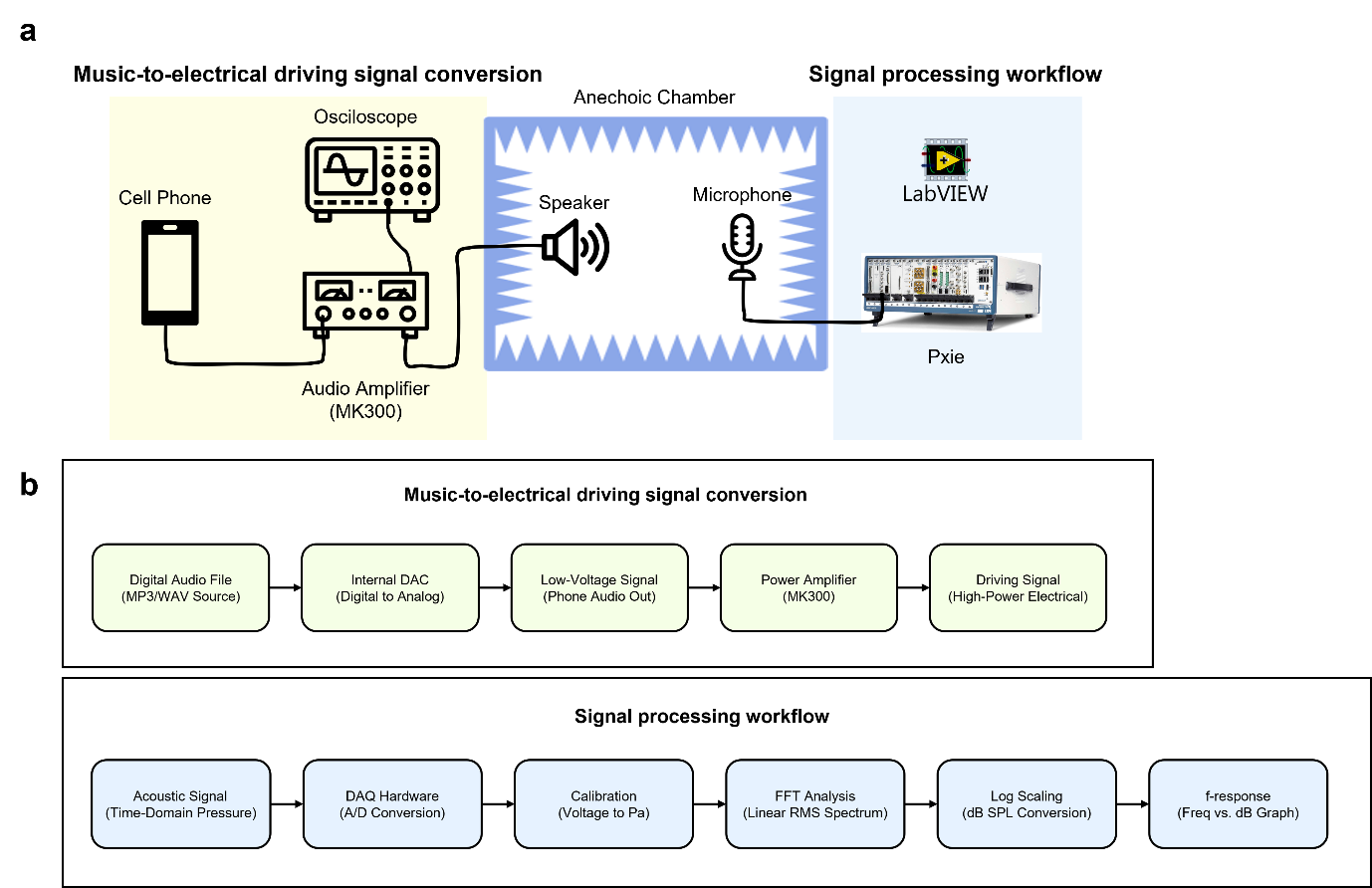


Figure S14. Measurement system and signal processing workflow for music-driven acoustic characterization of the OA-CLCEA. a) Schematic of the overall measurement setup. b) Detailed workflow of the music-to-electrical driving signal conversion (top) and acoustic signal processing (bottom).


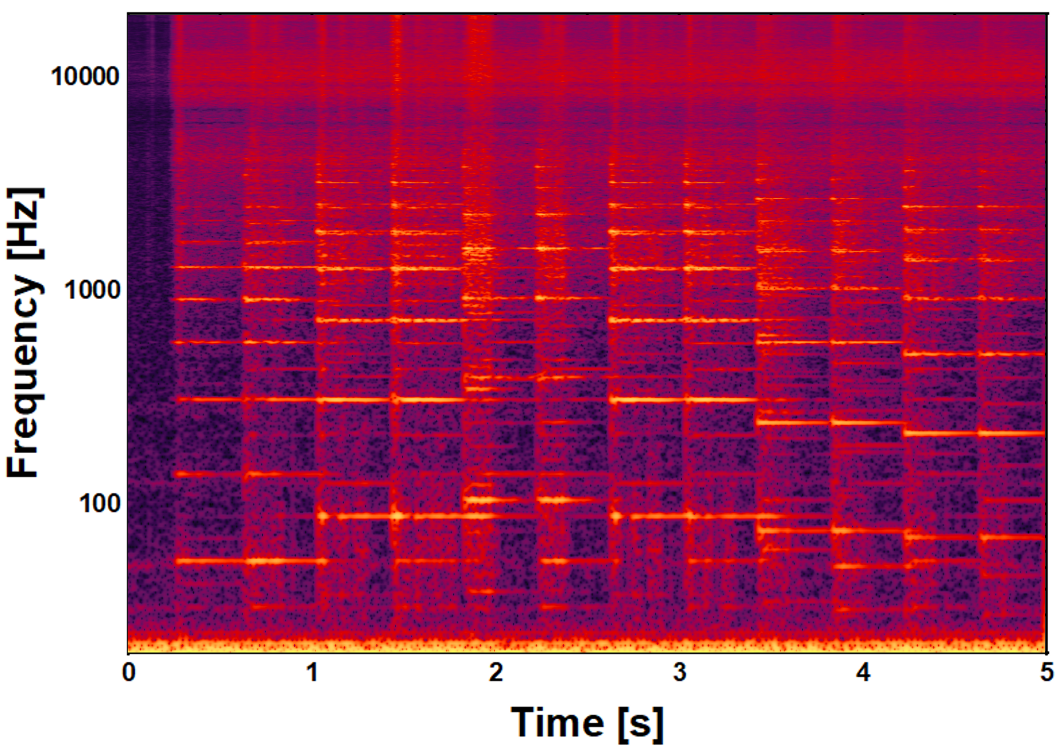


Figure S15. Spectrogram of music signal reproduced by the OA-CLCEA under DC electric field. Time-frequency representation of the acoustic output generated by the OA-CLEA when a music waveform (*“Twinkle, Twinkle, Little Star”* variations) is applied together with a DC electric field. Distinct frequency components corresponding to musical notes are observed, indicating accurate reproduction of the input music signal under DC-biased excitation.


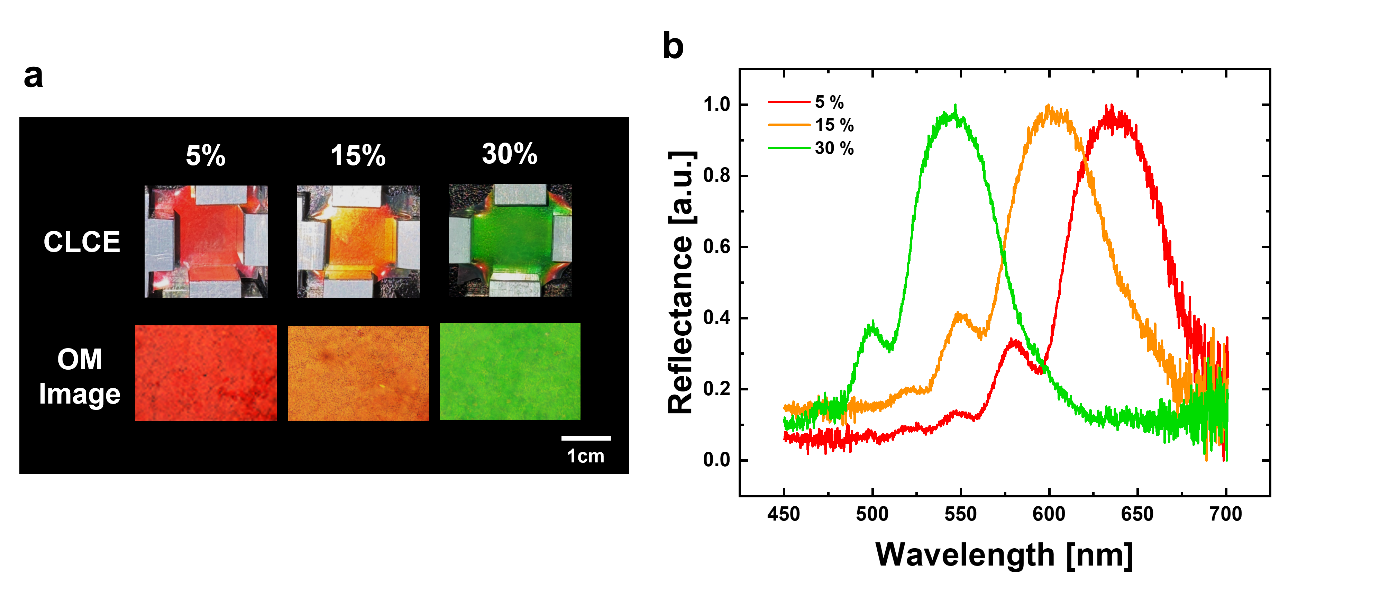


Figure S16. Structural-color change of the CLCE film under biaxial stretching.

a) Photographs and optical microscopy (OM) images of the CLCE film under biaxial strains of 5%, 15%, and 30%, showing a strain-induced blue shift in the reflected color. b) Reflection spectra of the biaxially stretched CLCE film, confirming the blue shift of the reflection band with increasing biaxial strain.


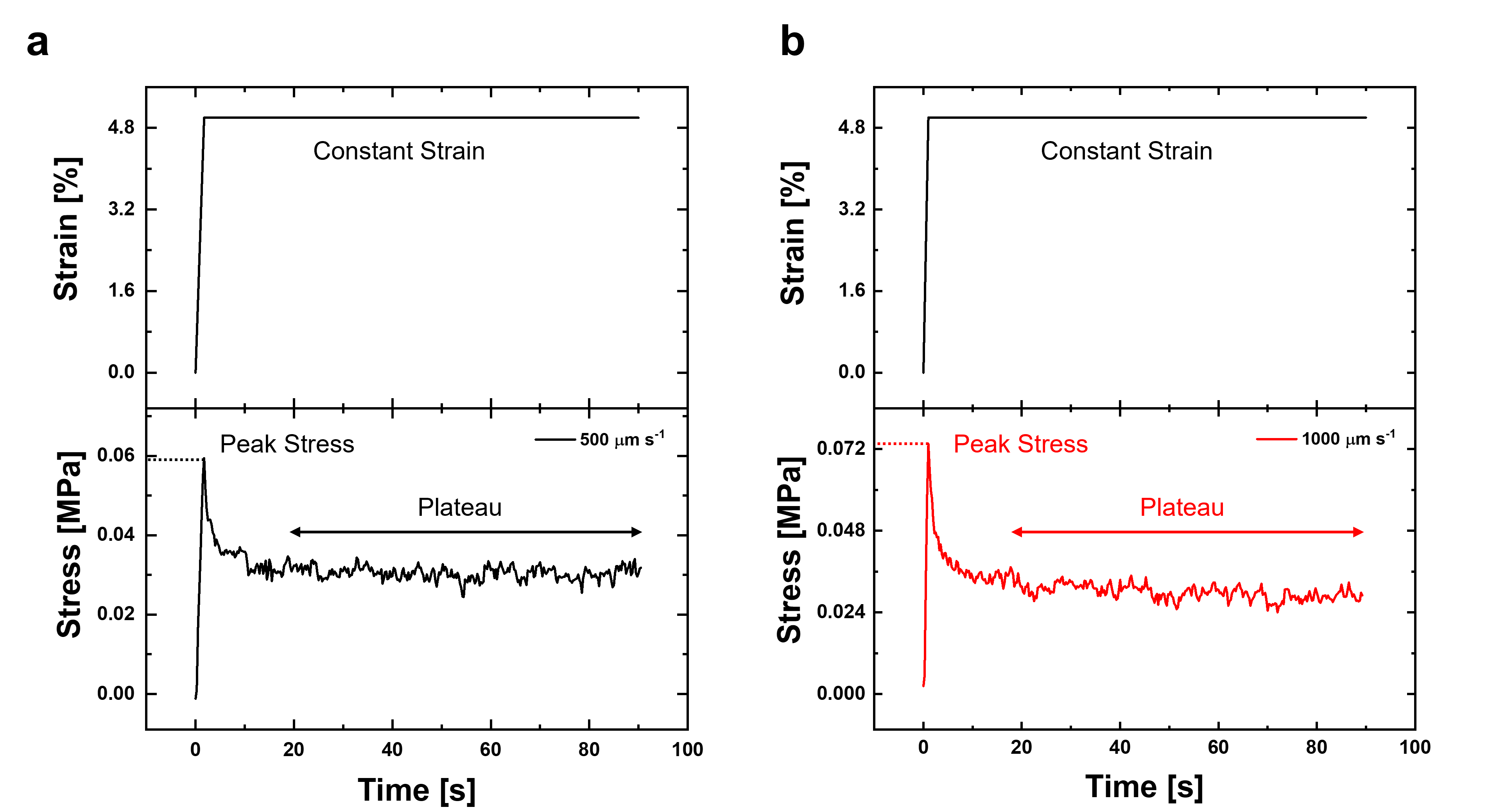


Figure S17. Stress relaxation behavior of the CLCE film under 5% constant strain.

a,b) Stress relaxation curves of the CLCE film after stretching to 5% strain at speeds of 500 μm s⁻¹ and 1000 μm s⁻¹, respectively. Although the stress relaxed after reaching the peak value, nonzero plateau stress remained in both cases, indicating that the CLCE film maintained a taut state under 5% pre-strain.

Supplementary Videos

**Video S1.** Electrochromic color switching of the OA-CLCEA driven by a low-frequency AC signal

**Video S2.** Electro-acoustic response of the OA-CLCEA under audible-frequency AC sweep

**Video S3.** Frequency-dependent electrochromic behavior of the OA-CLCEA under AC excitation

**Video S4.** Sequential operation of the OA-CLCEA showing the transition from acoustic only to optic-acoustic mode

**Video S5.** Independently tunable electro-opto-acoustic outputs in a monolithic OA-CLCEA

**Video S6.** Music reproduction by a monolithic OA-CLCEA under DC-biased complex signal excitation
